# Jiao Shao: A forerunner of physiological psychology and comparative psychology in China

**DOI:** 10.1007/s13238-018-0592-x

**Published:** 2018-11-16

**Authors:** Lijun Wang, Yanyan Qian, Yanjie Su

**Affiliations:** 1 School of Educational Science, Anhui Normal University, Wuhu, 241000 China; 2 Social and Behavioral Sciences Facility, Leiden University, PO Box 9555, 2300 RB Leiden, Netherlands; 3 School of Psychological and Cognitive Sciences and Beijing Key Laboratory of Behavior and Mental Health, Peking University, Beijing, 100871 China

**Figure 1:**
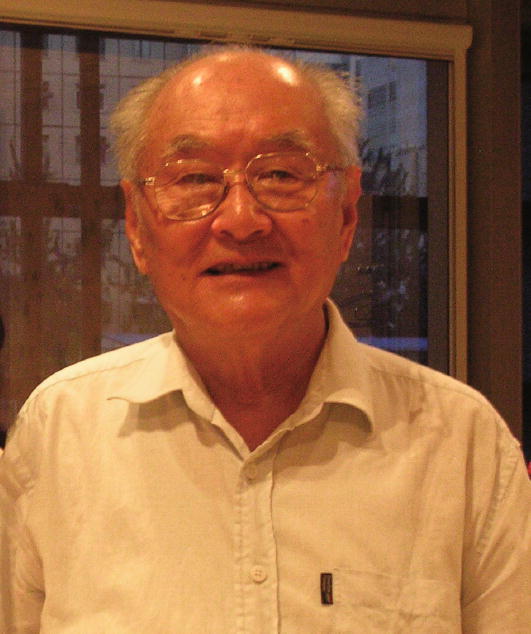
Jiao Shao (1923–2017)

Jiao Shao (邵郊, 1923–2017) (Fig. 1), who was an expert in physiological psychology and comparative psychology in China, made important contributions to the development of physiological psychology and comparative psychology, scientific research and personnel training (The obituary of Jiao Shao, 2017). He was a member of the Second Discipline Review Group of the State Council (Pedagogy), the director of the third and fifth sessions of Chinese Psychological Society (CPS), the chairman of the Professional Committee on Physiological Psychology of the CPS (1984–1988), an editorial advisory board member of the Encyclopedia of China Publishing House (First Edition), an editorial board member of Acta Psychologica Sinica (1979–1991), and he was honored as a fellow of the CPS (2007).

On June 27th, 1923, Shao was born in Jinan City, Shandong Province, where he lived until he entered the department of psychology at Tsinghua University in 1943. After graduating in 1948, he stayed at Tsinghua University to work as an assistant. Four years later, led by Kuohua Sun (孙国华), the deputy director of the philosophy department at Peking University, Shao joined this institute as Sun’s coadjutant. Shao assisted Sun by adjusting the division of disciplines; performing civil service; giving lectures; and completing experiments on higher nervous system activity, animal psychology, and comparative neuroanatomy (Dong, 1993; Qian and Li, 2011).

In the year of 1953, in collaboration with Naichang Shen (沈迺璋) and Kuohua Sun, Shao established the first national animal conditioned reflex laboratory (Qian and Li, 2011; Yan and Zhou, 2013), which, although it was later superseded by physiological psychology laboratory, was able to lay a strong foundation for psychology becoming a national key discipline (Newsroom of Acta Psychologica Sinica, 2017). This lab both helped to make clear the basic essence of Ivan Petrovich Pavlov’s experimentation and provided a test site to verity the physiological mechanism of conditioned reflexes (Zhang and Zhu, 1986; Editorial board of draft history of the philosophy department at Peking University, 2004). Under the special experimental conditions at that time, Shao published *The relationship between the frequency of electrical current and the motor effect when the dog*’*s sigmoid gyrus is under direct electrical stimulation* in a joint work with Naichang Shen and Techan Shen (沈德灿) (Shen et al., 1956). Shao’s lab later became a key state laboratory under the leadership of Shao (Newsroom of Acta Psychologica Sinica, 2017).

**Figure 2:**
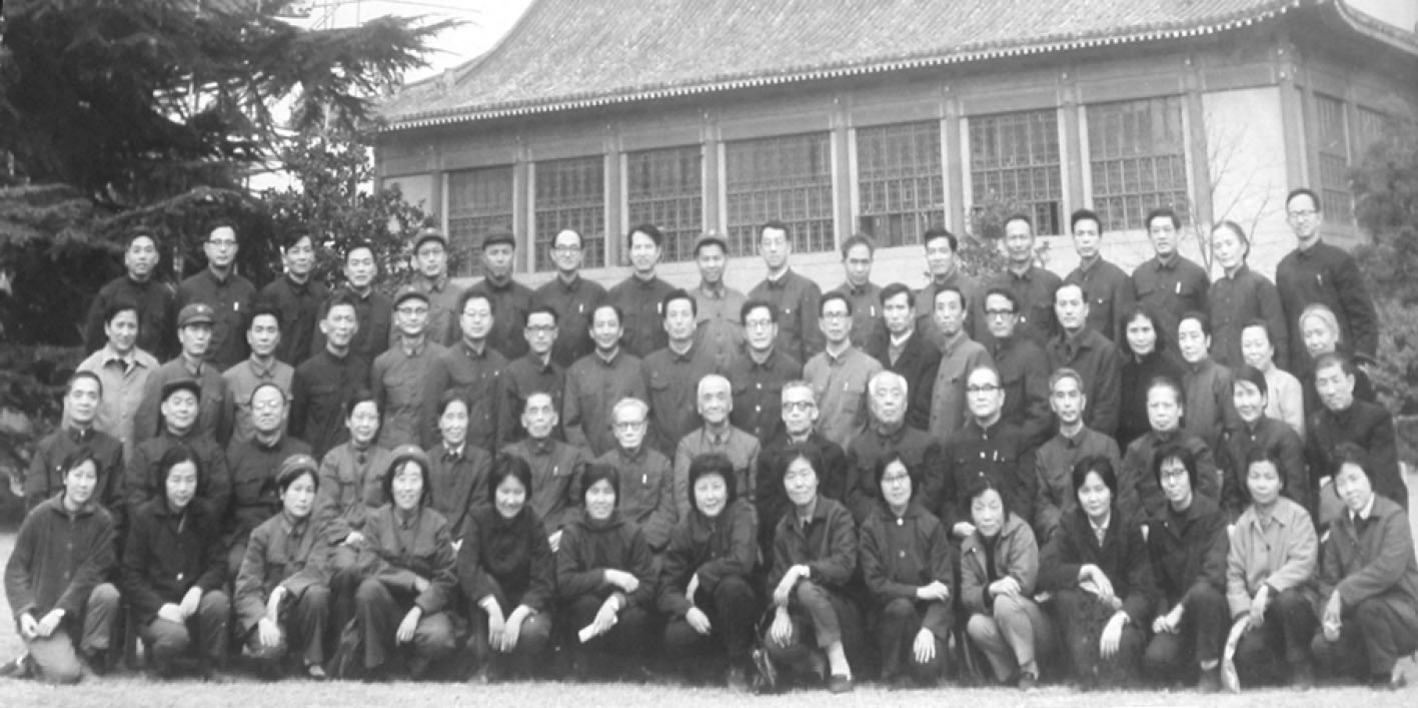
On November 20th, 1980, Jiao Shao (the fifth from right, in the second row) attended the national physiological psychology seminar at its inaugural meeting

**Figure 3:**
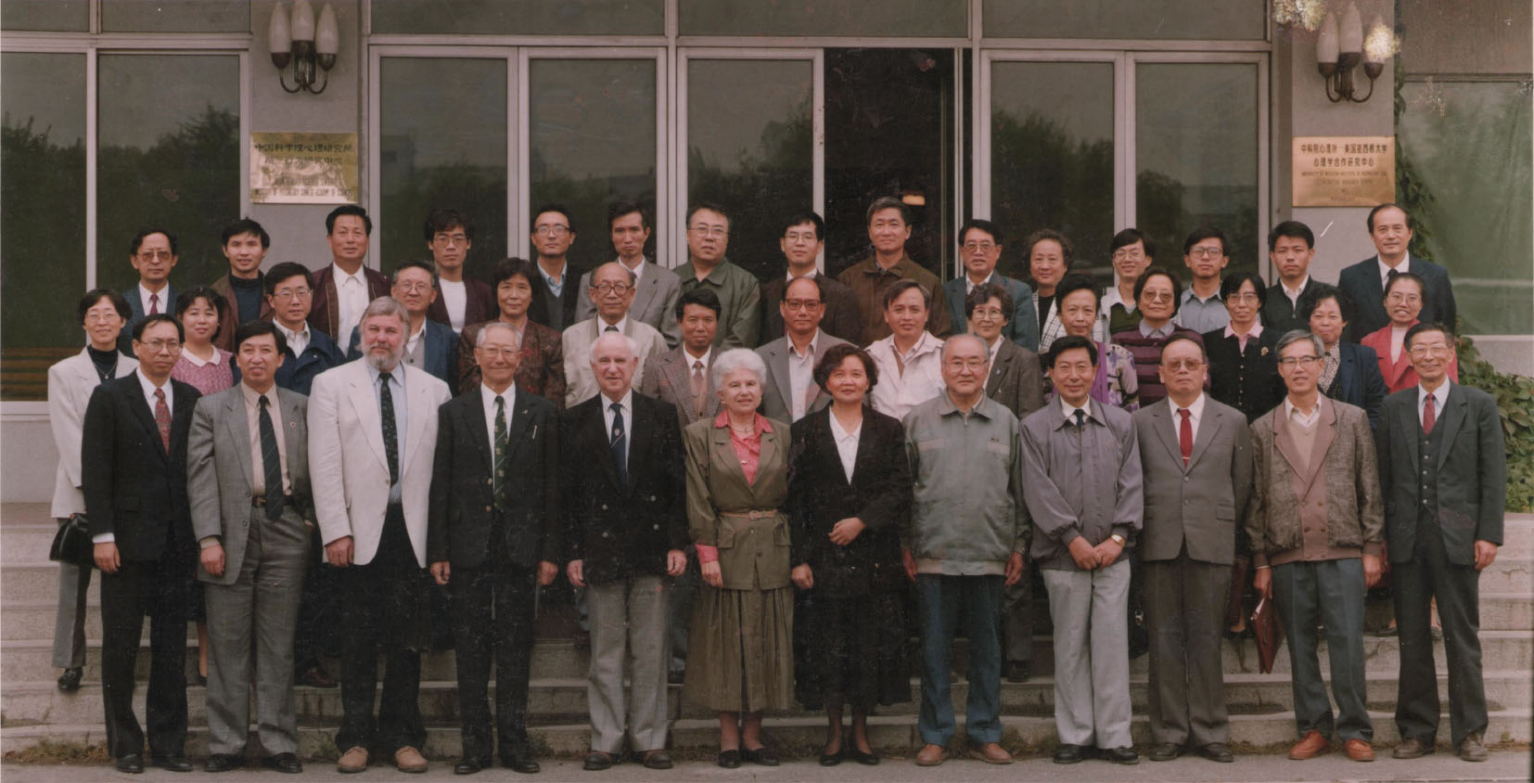
On October 10th, 1994, Jiao Shao (the first row, the fifth from right) attended the founding conference of the Center for Brain and Behavior Research in IPCAS

Shao worked at Peking University as an associate professor and then professor, starting in 1978 after the reconstruction of the department of psychology, until he retired. In 1975, Shao and Zhaolan Meng (孟昭兰) adopted the electric shock method to induce emotion and found the relationship between volume pulse, breathing and psychological stress (Shao and Meng, 1975). In 1981, Shao went to the University of Michigan to conduct a research program called *Long-term Inhibition of Kindled Seizures by Brain-Stimulation* for one year, where he cooperated with the world-renowned biopsychologist E. S. Valenstein. The results of this program were presented in *Experimental Neurology* (Shao and Valenstein, 1982). On November 20th, 1980, Shao attended the founding congress of the Professional Committee on Physiological Psychology of CPS at its first meeting (Fig. 2). On December 7th, 1981, Shao was elected as the director of CPS at the third session of CPS (Jing, 2001). In 1987, *The Physiological Psychology*, written by Shao, became a classic textbook and “Holy Writ” in the field of physiological psychology, and it is still in print today (Qian and Li, 2011).  On October 10th, 1994, Shao took part in the founding conference of the Center for Brain and Behavior Research in the Institude of Psychology, Chinese Academy of Science (IPCAS) (Fig. 3). Shao became one of the first members of psychologists assessed by CPS in 2009, and he was given the CPS Excellent Psychologist Award in 2016. Sadly, Shao passed away due to illness on September 18th, 2017.

In the field of comparative psychology, Shao guided numerous students in analyzing the evolution of lower creatures to higher animals from the perspective of phylogeny. The research objects included paramecia, earthworms, white mice, dogs and primates. Shao and his students hoped to find some important pieces of evidence for the theory of evolution from a psychological perspective (Qian and Li, 2011). For instance, Shao and other researchers observed the color light preference of the amphioxus in their paper “Examining the sensitivity of the amphioxus to monochrome lights of different wavelengths” (Shao et al., 1981). Their research showed that the focus of animal evolution is natural selection shaped by the tubular central nervous system (Shao et al., 1981). In addition, Shao also focused on animal learning to study the issue of evolution. Shao, Lin (林国彬), Wan (万传文) and Liu (刘范) explored the capacity of three golden-haired monkeys (*Rhinopithecus roxellana*e) for generalization ability from three dimensional objects to two dimensional representative photographs and pictures. However, this research found that all the subjects showed no generalization ability (Lin, et al., 1982). The researchers added children aged one-year-six-months to two-years-six-months as subjects, and found that they gained the generalization ability in parallel with their language development (Lin et al., 1981). According to Shao’s formulation, the evolution of animal intelligence is not an orthogenesis phenomenon, and a lack of language is one of the important reasons for why golden-haired monkeys do not have the capacity of generalization.

In 1998, Shao collaborated with Li in writing *Restricted lesions to ventral prefrontal subareas block reversal learning but not visual discrimination learning in rats*, which was published in *Physiology and Behavior*. It is worth noting that this paper has been cited 59 times and 2018 is the newest citation year (George et al., 2018). The results from this study indicated that “the mPFC of rats is not essential for discrimination learning, but that each of the 2 ventral subareas of the mPFC, PL and IL plays a critical role in reversal learning” (Li and Shao, 1998), which is inconsistent with the previous finding that “Extensive damage to the medial prefrontal cortex (mPFC) of rats causes reversal learning deficits” (Li and Shao, 1998).

In the field of physiological psychology, Shao mainly explored the neural mechanism of audio-epileptic seizures. Shao’s series of studies on rodents’ auditory epilepsy modal and kindling effect have had considerable influences internationally (Newsroom of Acta Psychologica Sinica, 2017). These experiments yielded some important results: First, it was found that the electrical brain stimulation of subcortical could not only cause the kindling effect, but also an inhibitory effect (Xue and Shao, 1983). Second, it was demonstrated that inferior colliculus is an important main center of auditory seizures by comparing electrical stimulation and an acoustic primed group (Xu and Shao, 1986). Third, they proved the role played by the inferior colliculus through experiments performed on audiogenic seizure susceptible rats (Hu and Shao, 1986).

**Figure 4:**
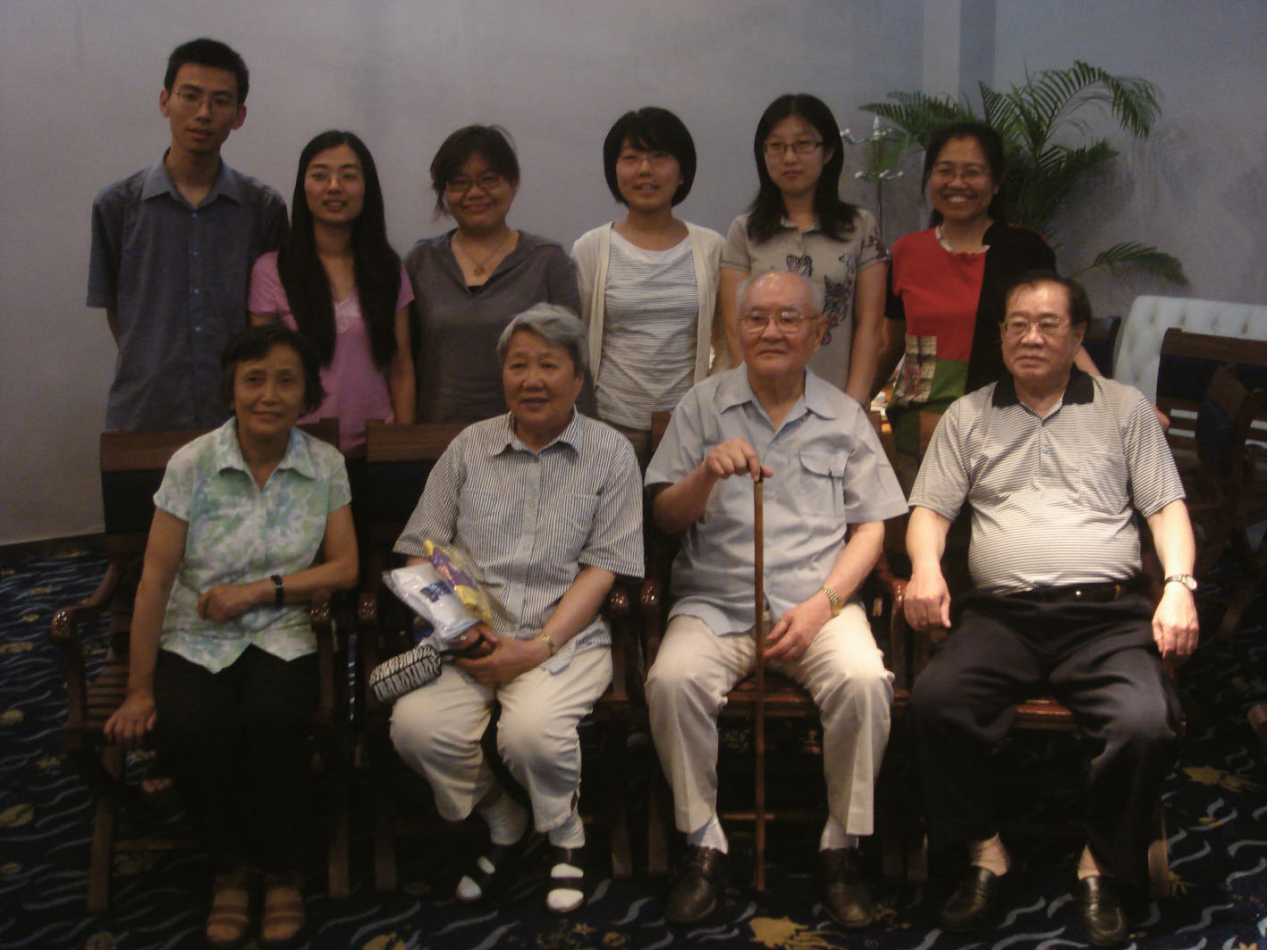
Jiao Shao and his young students

**Figure 5:**
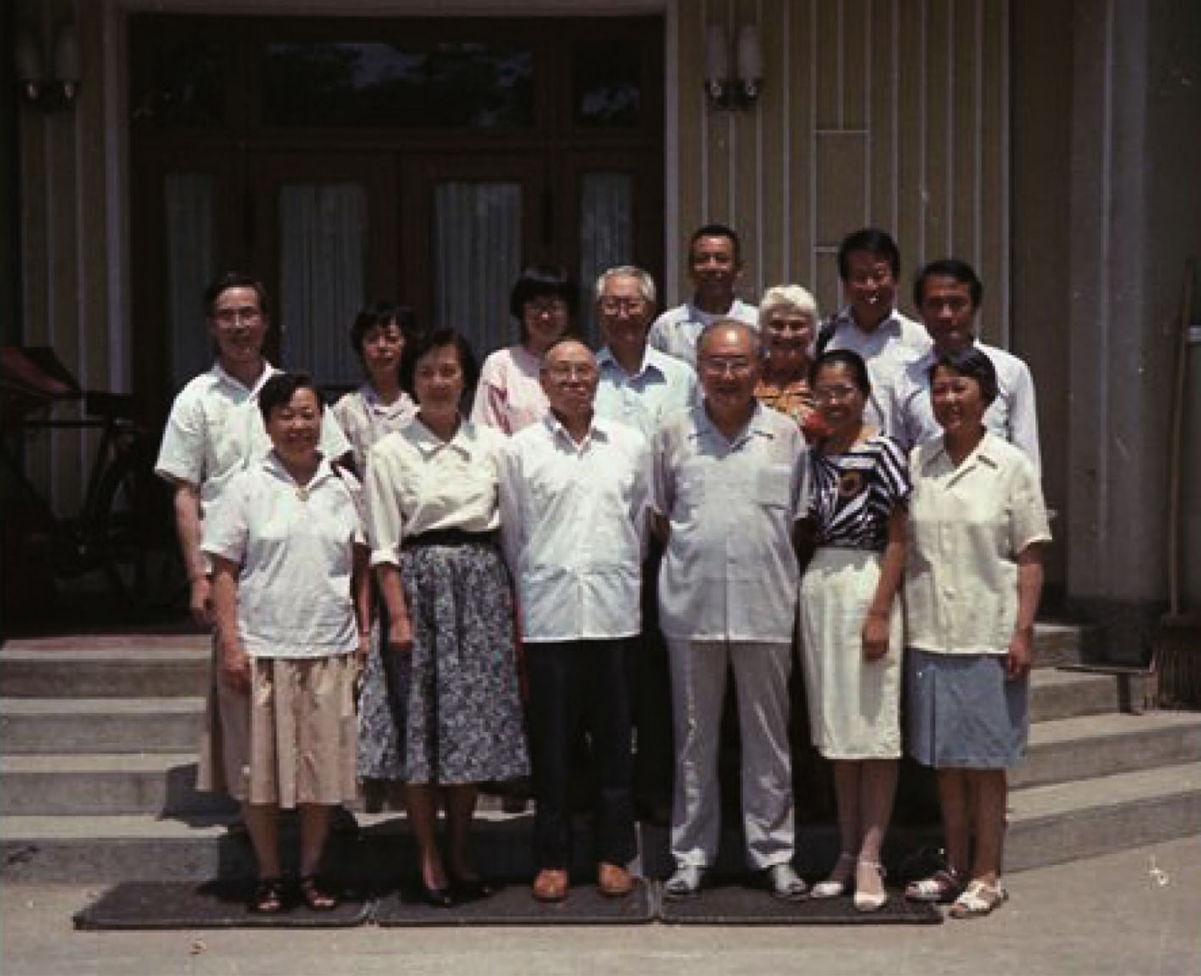
Jiao Shao (first row, third from right) at the doctoral dissertation defense of Yanjie Su (first row, second from right)

Shao devoted himself to the development of psychology, mainly focusing on disciplinary construction and personnel training. He translated some foreign classics with other scholars. One of these translations is *Principles of Neuropsychology*, written by A. R. Luria, and the other is *Comparative Psychology*—*A Modern Survey*, written by D. A. Dewsbury and D. A. Rethlingshafer, both of which were published by Sciences Press. Shao also offered guidance to many young scholars (Fig. 4). We retrieved the Wanfang Data and found that Shao mentored 15 students to finish PhD dissertations. For example, under Shao’s instruction, Yanjie Su (苏彦捷) (Fig. 5), who is now a professor at Peking University, analyzed macaca mulatta and macaca arctoides’s achievements in a delayed match-to-sample task (Su and Shao, 1992). Shao also led Xueming Zhang (张学明) and Bingqing Chen (陈炳卿) in carrying out a series of experiments to detect the relationship between Coca-Cola and auditory epilepsy (Zhang et al., 1985), and between Coca-Cola and movability behavior (Zhang et al., 1988).
